# Pregnancy outcome in delayed start antagonist versus microdose flare GnRH agonist protocol in poor responders undergoing IVF/ICSI: An RCT

**Published:** 2018-04

**Authors:** Robab Davar, Nosrat Neghab, Elham Naghshineh

**Affiliations:** 1 *Research and Clinical Center for Infertility, Yazd Reproductive Sciences Institute, Shahid Sadoughi University of Medical Sciences, Yazd, Iran.*; 2 *Department of Obstetrics and Gynecology, School of Medicine, Isfahan University of Medical Sciences, Isfahan, Iran.*

**Keywords:** Infertility, Assisted reproductive technology, Gonadotropins

## Abstract

**Background:**

Over the years, many article on different aspects of pathogenesis and management of poor ovarian responders have been published but there is no clear guideline for treating themyet.

**Objective:**

This study was designated to compare the effectiveness of a delayed start protocol with gonadotropin-releasing hormone (GnRH) antagonist and microdose flare-up GnRH agonist protocol in poor ovarian responders.

**Materials and Methods:**

This randomized clinical trial consisted of 100 poor ovarian responder women in assisted reproductive technologies cycles. They were divided randomly in delayed-start antagonist protocol (with estrogen priming followed by early follicular-phase GnRH antagonist treatment for 7 days before ovarian stimulation) and microdose flare-up GnRH agonist protocol. The main outcome was clinical pregnancy rate and second outcome was the number of retrieved oocytes, mature oocytes, 2PN number, fertilization rate, and implantation rate.

**Results:**

Fertilization rate, clinical pregnancy rate, and ongoing pregnancy rates were not significantly different between the two studied protocols. Number of retrieved oocytes (5.10±3.41 vs. 3.08±2.51) with p=0.002, mature oocytes (4.32±2.69 vs. 2.34±1.80) with p=0.003, number of 2PN (3.94±1.80 vs. 2.20±1.01) with p=0.001 and implantation rate (19.40% vs. 10.30%) with p=0.022 were significantly higher in delayed antagonist group.

**Conclusion:**

The delayed-start protocol can improve ovarian response in poor responders by stimulating and synchronizing follicle development.

## Introduction

Age-related infertility is gradually increasing and the subsequent demand for assisted reproductive technologies (ART) is increasing (1). The prevalence of poor ovarian responders (POR) in ART cycles varies from 5.6-35.1% depending on the classification of POR. Regardless of the definition and classification, a poor ovarian response to control ovarian hyper stimulation (COH) potentially results in high cycle cancellation rates, reduced numbers of retrieval oocytes, decreased numbers of transferred embryos, and lower pregnancy rates compared with normal ovarian response (2).

Several COH protocols have been described for handling POR but currently, there are no clear methods and guidelines for handling these women (3). Bologna criteria in 2011 published by the European Society of Human Reproduction and Embryology to standardize the definition POR in a simple way. POR is defined when at least two of the following three criteria is present:” (1) Maternal age >40 yr or any other risk factor for POR. (2) A previous POR (≤3 oocytes with a conventional stimulation protocol). (3) An abnormal ovarian reserve test [i.e. antral follicle count <5-7 follicles or anti-Müllerian hormone below 0.5-1.1 ng/ml” (1).

Several strategies have been suggested for POR. One of the most frequently used COH protocols is beginning of gonadotropin and a gonadotropin-releasing hormone (GnRH) agonist together in the follicular phase and so called microdose flare protocol (4). The merits of this protocol are an initial rise in endogenous gonadotropin and prevention in premature luteinizing hormone surge. Pretreatment with oral contraceptive pills prevents corpus luteum formation (5, 6). 

In the current study, we hypothesized that by postponing the start of COH with GnRH antagonist pretreatment for 7days after estrogen priming; there would be additional suppression of endogenous follicle-stimulating hormone (FSH) during the early follicular phase, resulting in more FSH-responsive follicles and therefore better synchronization of follicular growth. To investigate this hypothesis, we compared the COH outcomes of delayed start antagonist protocol with microdose flare GnRH agonist protocol.

## Materials and methods

This study was a randomized controlled trial including 100 POR women who were a candidate for in vitro fertilization/ intra cytoplasmic sperm injection between October 2016 and December 2016. According to the European Society of Human Reproduction and Embryology agreement, at least two of the following three features indication must be present to define POR: 

1) Advanced maternal age (≥40) or any other risk factor for the poor ovarian response, 

2) Previous poor response (cycles canceled or ≤3 oocytes with a conventional stimulation protocol), 

3) Abnormal ovarian reserve test (ORT) (AMH <0.5-1.1 ng/mL or AFC <5-7 follicles) (1). Patients with POR who underwent COH with a delayed start protocol with GnRH antagonist or microdose flare-up GnRH agonist protocol were chosen for this study.

Patients with age <18 or >42 yr, BMI ≥30 kg/m^2^, endocrine or metabolic disorders, severe endometriosis and severe male factor (patients with azoospermia and frozen-thawed sperms) were excluded from the study. The patients were randomly (Random Digit Software) allocated to two equal sized groups. Fifty women in group I received delayed start protocol with GnRH antagonist (delay protocol) and 50 women in group II received microdose GnRH agonist flare (microdose protocol).

**Treatment protocols**

Baseline ultrasound on cycle day 2 was performed to document the absence of ovarian cyst in two groups. In group I, 2 mg estradiol (Aburaihan Pharmaceutical Co., Tehran, Iran) was started orally twice a day on the 21^st^ luteal day and continued for 10 days. From 2^nd^ day of cycle GnRH antagonist pretreatment (0.25 mg Cetrotide, Merck-Serono Germany) was started and continued for 7 days then ovarian stimulation initiated with 300 IU FSH (Gonal-F, Serono, Italy) and then Cetrotide 0.25 mg added again if the ultrasound monitoring showed at least one follicles with diameter ≥14 mm to prevent premature ovulation and was continued until the trigger day.

In group II, low-dose contraceptive pills (30 mcg Ethinyl Estradiol and 0.3 mg Norgestrel, Aburaihan Pharmaceutical Co., Tehran, Iran) was initiated on the 2^nd^ day of the previous cycle for 21 days, after withdrawal bleeding on the second day of menstruation, Suprefact (Buserelin acetate, Aventis Pharma Deutschland, Germany) 50 mcg SC twice a day was started and continued until the day of Human chorionic gonadotropin (hCG) administration. After 2 days (on the fourth day of menstruation), the patients received Gonal-F 300 IU/day. In these patients, like in the other group, the dose of Gonal-F was adjusted according to serum estradiol concentrations and ovarian responses as noted by ultrasound.

In both groups, 10,000 IU of hCG (Pregnyl, Daropakhsh, Iran) was administered IM when at least three follicles reached ≥17 mm in diameter. After 36 hr, ultrasound-guided transvaginal oocyte retrieval was performed and the follicles ≥14 mm was aspirated while the physicians performing the follicular aspiration were blinded to the stimulation protocol. The in vitro fertilization and intra cytoplasmic sperm injection procedures were performed, and the embryos were transferred on the third day after retrieval with a Labotect catheter (Labotect, Gotting, Germany). The criteria for embryo quality were used from the embryo morphology assessment according to Dokras and co-workers.

The cleavage-stage embryos scored as grade A, B, C, and D. Grade A embryo: no fragmentation with equal sized homogenous blastomeres, grade B embryo: <20% fragmentation with equal sized homogenous blastomeres, grade C embryo: 20-50% fragmentation with unequal sized blastomeres, grade D embryo: >50% fragmentation with unequal sized blastomeres (7). The grade D embryos were not transferred.

The number of transferred embryos depended on the embryo quality and the patient’s age. All the patients received 400 mg of suppository progesterone (Cox Pharmaceuticals, Barnstaple, UK) twice a day for luteal support, which was initiated on the day of oocyte retrieval. Fourteen days after the embryo transfer Serum β-hCG was checked. If pregnancy occurs, then progesterone will continue until the 10^th^ wk of pregnancy. 

Ratio of gestational sacs to the number of embryos transferred is defined as implantation rate, β-hCG ≥50 IU/L after 14 days from embryo transfer and presence of a gestational sac with heartbeat identified by ultrasound 5 wk after the embryo transfer are classified as chemical and clinical pregnancy respectively. Pregnancy loss before 20 wk of gestation and pregnancy continued after 20 wk categorized as abortion and ongoing pregnancy respectively.

**Outcome measures**

The primary outcome was clinical pregnancy rate. While secondary outcomes were; total dosage of gonadotropin, the duration of stimulation, the number of retrieved oocytes, embryo yield, the endometrial thickness in triggering day, implantation rate, ongoing pregnancy and abortion rate.

**Ethical consideration**

The study was approved by Ethics Committee of Research and Clinical Center for Infertility, Shahid Sadoughi University of Medical Sciences (IR.SSU.RSI.REC.1395.3). A written informed consent was obtained from all patients.

**Statistical analysis**

The Statistical Package for Social Sciences (SPSS, version 16.0, SPSS Inc, Chicago) was used to perform all the statistical analyses. The Chi square (χ^2^) test was used to analyze nominal variables. Normally distributed Kolmogorov-Smirnov test parametric variables were tested by independent Student’s t-test. Non normally distributed metric variables were analyzed by Mann-Whitney U test. p<0.05 was considered statistically significant. Values were expressed as mean±SD unless otherwise stated.

## Results

One hundred twelve patients were enrolled in this study, and 12 of them were declined to participate; therefore, the final analysis was done on 100 POR women, 50 were treated with a delayed protocol, and 50 received microdose protocol (Figure 1).

Baseline characteristics of the patients included in the study are presented in table I. There was no significant difference in demographic characteristics. Table II compares the cycle characteristics in the two groups. Endometrial thickness in trigger day, number of total and mature oocytes (MII) retrieved and numbers of 2 pro-nuclei (2PN) were significantly higher in the delayed group but total dose of gonadotropins was significantly higher in microdose group. 

The embryo data and the clinical outcome of the study groups are compared in table III. Implantation rate was significantly higher in the delayed group but fertilization rate, clinical pregnancy rate per cycle and ongoing pregnancy rate had no significant difference.

**Table I:** Demographic and infertility characteristic of patients

**Variable**	**Delay antagonist protocol**		**Microdose protocol**		**p-value**
	**Mean ± SD**	**Median**	**Mean ± SD**	**Median**	
Age (yr)	38.38 ± 2.50	38.50	0.511	39.00	0.511
Day 3 FSH level (mIU/mL)	7.84 ± 2.02	7.80	0.879	7.80	0.879
Duration of infertility (yr)	6.67 ± 5.15	5.00	0.207	4.00	0.207
AMH	0.75 ± 0.48	0.70	0.066	0.90	0.066
AFC	4.88 ± 1.99	5.00	0.581	5.00	0.581
Estradiol level on hCG day (pg/mL)	1203 ± 728.12	989.50	0.553	1210.00	0.553
Progesterone level on hCG day (pg/mL)	0.79 ± 0.56	0.65	0.108	0.50	0.108

p-value <0.05 was significant (Student t-test)

FSH: Follicular stimulating hormone

AMH: Anti mullerian hormone

AFC: Antral follicular count

hCG: Human chorionic gonadotropin

**Table II:** Cycle characteristics of the patients

**Variable**	**Delay antagonist protocol**		**Microdose protocol**		**p-value**
	**Mean ± SD**	**Median**	**Mean ± SD**	**Median**	
Days of ovarian stimulation*	14.24 ± 2.15	15.00	12.82 ± 1.48	13.00	0.060
Total dose of gonadotropins (IU)$	3223.50 ± 900.19	3000.00	3604.50 ± 786.22	3750.00	0.000
Endometrial thickness (mm) in trigger day$	10.44 ± 2.15	10.00	9.10 ± 1.71	9.00	0.001
Total oocytes retrieved$	5.10 ± 3.41	4.00	3.08 ± 2.51	2.50	0.002
MII oocytes retrieved$	4.32 ± 2.69	4.00	2.34 ± 1.80	3.00	0.003
NO.2 PN$	3.94 ± 1.80	2.00	2.20 ± 1.01	2.00	0.001
Total embryo NO*	1.74 ± 1.73	1.00	1.62 ± 0.532	1.00	0.190
Transfer embryo NO*	1.34 ± 1.09		1.08 ± 0.90		
Embryo quality grade A# B# C#	5 (13.5%) 24(64.9%) 6 (16.2%)	1.00	6 (14.00%) 30 (69.8%) 7 (16.3%)	1.00	0.242 0.494

Data are presented as mean±SD. p-value <0.05 was significant

* Student t test

$ Mann-Whitney U test

# Chi square (χ2) test

**Table III:** Embryo data and clinical outcome of the patients

	**Delay antagonist protocol**	**Microdose protocol**	**p-value**
Fertilization rate	59.7%	56.6%	0.49
Implantation rate	19.40%	10.30%	0.022
Clinical pregnancy rate#@	9 (18.00%)	7 (14.00%)	0.91
Clinical abortion rate#@	2 (22.00%)	1 (14.00%)	0.88
Ongoing pregnancy rate#@	7 (14.00%)	6 (12.00%)	0.102

@ Data are presented as n(%). p-value <0.05 was significant

# Chi square (χ2) test

**Figure 1:**
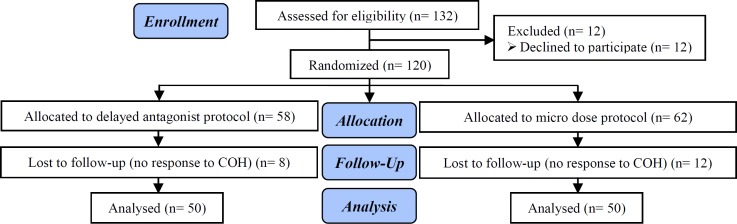
Consort flow chart

## Discussion

There were many attempts to improve ART outcome in POR. In infertile patients, an increase in the number of oocytes and embryos is a critical aspect of a successful cycle (8). In the current study, we applied a protocol combining estrogen priming in the prior luteal phase and a short pituitary down regulation with GnRH antagonist in the early follicular phase immediately, followed by COH and compared with microdose protocol. Delayed protocol had a statistical difference in oocytes retrieved, mature oocyte yield, 2 PN number in POR compared with microdose protocol. 

A better understanding of follicular growth has resulted in the development of policies for ovarian stimulation in POR. It has been found that during the luteal-follicular transition, FSH protects early antral follicles from atresia and causes their growth. Larger follicles are more sensitive to FSH and, as a result, begin to respond and develop during the late luteal phase. Therefore, asynchronous follicular growth during COH may be a consequence of the size heterogeneities of early antral follicles during the early follicular phase (9).

Coordinated Follicular growing is required to response to COH to achieve simultaneous maturation in ART cycles. Noticeable discrepancies in follicular size result in diminished number of oocyte maturation and fertilization potential (10). In our study estradiol priming and GnRH, antagonist pretreatment may be resulted in more synchronous follicular growth and better ART results. Until now, few studies have evaluated whether a delayed protocol improves COH outcomes.

Endogenous FSH may stimulate larger follicles in the prior luteal phase selectively and subsequently lead to a size discrepancy in the developing follicles. Fewer follicles may be a response to COH due to this size inconsistency (11).

Fanchin and colleagues found that estradiol priming in comparison with a control group resulted in a more number of follicles larger than 16 mm, further mature oocytes, and more embryos (12). Frankfurter and coworkers showed in POR, pretreatment with GnRH antagonist on cycle day 5-8 with two doses of cetrorelix acetate (3 mg) 4 days apart with daily progestin were used to extend the oocyte recruitment interval. This protocol improved numbers of retrieval oocytes and transferred embryos (13). In our trial, the length of GnRH antagonist pretreatment was 7 days and we started Cetrotide on cycle day 2, after estrogen priming to suppress early follicular phase FSH rise.

In another clinical trial performed by Younis and coworkers among normal responders, 3-day GnRH antagonist pretreatment before COH in an antagonist protocol improved oocyte maturity and fertilization rates but did not change the pregnancy rates (14). Blockeel and colleagues in a pilot study among women with normal ovarian reserve showed that early follicular phase GnRH antagonist pretreatment for 3 days resulted in a trend toward a higher number of retrieved oocytes but failed to yield significantly higher pregnancy rates as in our results in POR (15).

Cakmak and coworkers in a retrospective study compared delayed protocol with conventional antagonist protocol. In their study as in our research, delay protocol resulted in more synchronous follicle growth, higher mature oocyte yield, and more embryos to transfer compared with conventional estrogen priming GnRH antagonist protocol (2). Aflatoonian and colleagues in a clinical trial concluded that in poor responders delayed-start protocol slightly but not significantly improves pregnancy and implantation rate. They compared conventional antagonist protocol with delayed-start protocol (16). In our study delayed protocol was compared with microdose flare to find a suitable ART protocol in poor responders and the results indicated that the number of retrieved oocytes, mature oocytes, number of 2 PN and implantation rate were significantly higher in delayed antagonist group. 

We assume that by suppressing the endogenous FSH and providing a hormonal environment for the follicles to express similar amounts of FSH receptors and consequently COH with more synchronous follicular growth and better ART results.

## Conclusion

It may be possible by using of the delayed protocol improve ovarian responsiveness during COH that result to more uniform follicular development, more mature oocytes retrieved, transfer of higher numbers of embryos, and possibly improved pregnancy rates compared with microdose protocol in POR. Although this treatment protocol had longer stimulation period, it gives new hope to POR.

## Note

***Registration ID in IRCT: IRCT2016071628950N1***
